# Structural basis for double-stranded RNA recognition by SID1

**DOI:** 10.1093/nar/gkae395

**Published:** 2024-05-14

**Authors:** Runhao Wang, Ye Cong, Dandan Qian, Chuangye Yan, Deshun Gong

**Affiliations:** State Key Laboratory of Medicinal Chemical Biology and College of Life Sciences, Nankai University, Tianjin 300350, China; School of Life Sciences, Tsinghua University, Beijing, 100084, China. Tsinghua-Peking Joint Center for Life Sciences, Tsinghua University, Beijing, 100084, China. Beijing Frontier Research Center for Biological Structure, Beijing Advanced Innovation Center for Structural Biology, Tsinghua University, Beijing, 100084, China. State Key Laboratory of Membrane Biology, Tsinghua University, Beijing 100084, China; State Key Laboratory of Medicinal Chemical Biology and College of Life Sciences, Nankai University, Tianjin 300350, China; School of Life Sciences, Tsinghua University, Beijing, 100084, China. Tsinghua-Peking Joint Center for Life Sciences, Tsinghua University, Beijing, 100084, China. Beijing Frontier Research Center for Biological Structure, Beijing Advanced Innovation Center for Structural Biology, Tsinghua University, Beijing, 100084, China. State Key Laboratory of Membrane Biology, Tsinghua University, Beijing 100084, China; State Key Laboratory of Medicinal Chemical Biology and College of Life Sciences, Nankai University, Tianjin 300350, China

## Abstract

The nucleic acid transport properties of the systemic RNAi-defective (SID) 1 family make them attractive targets for developing RNA-based therapeutics and drugs. However, the molecular basis for double-stranded (ds) RNA recognition by SID1 family remains elusive. Here, we report the cryo-EM structures of *Caenorhabditis elegans* (c) SID1 alone and in complex with dsRNA, both at a resolution of 2.2 Å. The dimeric cSID1 interacts with two dsRNA molecules simultaneously. The dsRNA is located at the interface between β-strand rich domain (BRD)1 and BRD2 and nearly parallel to the membrane plane. In addition to extensive ionic interactions between basic residues and phosphate backbone, several hydrogen bonds are formed between 2′-hydroxyl group of dsRNA and the contact residues. Additionally, the electrostatic potential surface shows three basic regions are fitted perfectly into three major grooves of dsRNA. These structural characteristics enable cSID1 to bind dsRNA in a sequence-independent manner and to distinguish between DNA and RNA. The cSID1 exhibits no conformational changes upon binding dsRNA, with the exception of a few binding surfaces. Structural mapping of dozens of loss-of-function mutations allows potential interpretation of their diverse functional mechanisms. Our study marks an important step toward mechanistic understanding of the SID1 family-mediated dsRNA uptake.

## Graphical Abstract

**Graphical Abstract:**
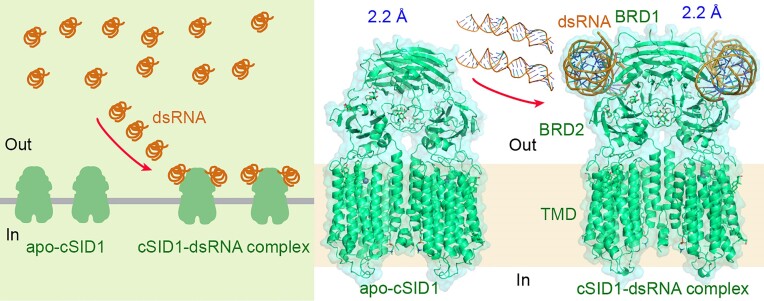

## Introduction

Systemic RNA interference (RNAi) is the term used to describe the still-mysterious ability of *Caenorhabditis elegans* to disperse the silencing RNA from an original place to adjacent tissues and cell progeny (1). The Craig P. Hunter group carried out groundbreaking research in this area. They first discovered that the systemic RNAi-defective protein 1 (SID1) acts as a double-stranded RNA (dsRNA) channel, allowing dsRNA to enter cells without the need for energy (2,3). As extracellular RNAs plays vital roles in cell-cell communications, virus-host interactions and potent RNA therapeutics in mammalian body (4,5), the two mammalian orthologs of *Caenorhabditis elegans* SID1 (cSID1), SID transmembrane family member 1 (SIDT1) and SIDT2, have been receiving increasing attention in serving as potential targets for the development of RNA-based therapeutics and drugs.

MicroRNA-21, an ‘oncogenic’ miRNA, is widely overexpressed in human cancer and promotes therapeutic resistance in a number of human cancers (6). Human SIDT1 (hSIDT1) mediates contact-dependent small RNA transfer and microRNA-21-driven chemoresistance (7), suggesting that it may be a tractable target for novel therapies aimed at improving the efficacy of current cytotoxic agents (7). SIDT1 partially localizes to endolysosomes and transports internalized dsRNA into the cytosol for antiviral immunity (8). Intriguingly, SIDT1 expressed on gastric pit cells in the stomach mediates host uptake of dietary and orally administered microRNAs, shedding light on developing small RNA therapeutics by oral delivery in the future (9). Similarly, SIDT2 also localizes to endolysosomes and transports extracellular dsRNA into the cytoplasm for innate immune recognition (10). SIDT2 predominantly localizes to lysosomes and mediates direct uptake of RNA and DNA by lysosomes for degradation, a novel type of autophagy named RN/DNautophagy (11,12). These nucleic acid transport properties make them attractive candidates for the development of nucleic acid therapeutics.

To reveal the transport properties of SID1 family proteins, Craig P. Hunter and coworkers found that cSID1 is not selective for dsRNA length and the import rates of dsRNA are dependent on dsRNA concentration (13). Furthermore, they found that cSID1 is a dsRNA-selective dsRNA-gated channel in *Drosophila S2* cells, indicating that cSID1 can discriminate between dsRNA and dsDNA under an unclear substrate recognition mechanism (14). Instead, Jae Man Lee group found that cSID1 is competent for plasmid DNA uptake in *silkworm BmN4* cells (15). Min Li group revealed that the recombinant purified cSID1 extracellular domain (ECD) selectively binds dsRNA but not dsDNA (with a lower binding affinity) in a length-dependent and sequence-independent manner (16). Mouse SIDT1 and SIDT2 ECDs bind to dsRNA with apparent lower affinities than that of cSID-1 ECD (16). In addition, the cytosolic domain of SIDT2 carries an arginine-rich motif also binds to both DNA and RNA, and is important for the direct transport of nucleic acids into lysosomes (17). Recently, several studies reported that mammalian SIDT1 and SIDT2 can also mediate single-stranded RNA uptake (9,18). However, the mechanisms of nucleic acid recognition and transport by SID1 family proteins remain extremely elusive, a question that has puzzled scientists for more than two decades.

Besides the roles in diverse nucleic acid transport processes, the member of SID1 family proteins, SIDT2, also plays essential roles in regulating glucose metabolism (19), lipid metabolism (20–22), skeletal muscle homeostasis (23), lung and gastrointestinal tumor development (24), and cholesterol transport (25). Recently, we report the atomic structure of human SIDT2, the first structure of SID1 family proteins, revealing that no discernible nucleic acid conduction pathway has been identified within the transmembrane domain (TMD) or the interface of the dimeric TMDs under detergent micelles condition, suggesting that SIDT2 may function as a transporter rather than a channel (26). Intriguingly, we found that hSIDT2 can hydrolyze C18 ceramide into sphingosine and fatty acid with a slow rate by a zinc-dependent catalytic core within the TMD (26), which is the first evidence that SID1 family proteins have an intramembrane hydrolase activity. Although a large number of positively charged residues are found on both β-strand rich domain 1 (BRD1) and BRD2 in the extracellular side, no clear density of RNA was found in the structure of hSIDT2 in complex with a 20-nt (nucleotides) single-stranded microRNA in a low-pH environment (26).

Due to the lack of systemic RNAi in human and the low sequence identity between cSID1 and hSIDT2, indicating that they have respective unique features in structure and function. In this study, we report the cryo-EM structures of full-length cSID1 alone and in complex with a 50-base pairs (bp) dsRNA, revealing the mechanism of dsRNA recognition by cSID1.

## Materials and methods

### Transient protein expression and purification

The full-length *C. elegans* SID1 cDNA was subcloned into the pCAG vector with a His_6_-tag and a FLAG-tag following the signal peptide. HEK293F cells (Invitrogen) were cultured in SMM 293T-II medium (Sino Biological Inc.) at 37°C under 5% CO_2_ in a Zhichu shaking incubator (ZQWY-AS8E, 120 rpm). When the cell density reached 2.0 × 10^6^ cells/ml, the pCAG-cSID1 plasmids were transiently transfected into the cells. For 1-l HEK293F cell culture, approximately 2 mg of plasmids were pre-mixed with 4.0 mg 25-kDa linear polyethylenimines (PEIs) (Polysciences) in 50 ml fresh medium for 20–30 min before transfection. The 50 ml mixture was then added to the cell culture. The transfected cells were cultured for 48 h before harvesting.

For purification, 8-l HEK293F cells were harvested by centrifugation at 800g for 10 min and resuspended in the lysis buffer containing 25 mM HEPES (pH 7.4), 150 mM NaCl (lysis buffer A), 2.6 μg/ml aprotinin, 4 μg/ml pepstatin, and 2 μg/ml leupeptin. The lysate was incubated in the buffer containing 1% *n*-dodecyl-β-d-maltopyranoside (DDM, Anatrace) plus 0.1% cholesteryl hemisuccinate tris salt (CHS, Anatrace) at 4°C for 2 h for membrane protein extraction. After ultracentrifugation at 18 700g for 1 h, the supernatant was collected and applied to the anti-FLAG M2 affinity gel (Sigma) at 4°C for two time. The resin was washed six times with 5 ml wash buffer A (lysis buffer A plus 0.02% glycol-diosgenin (GDN, Anatrace)) each time. The protein was eluted with elution buffer A (wash buffer A plus 300 μg/ml FLAG peptide (GL Biochem). The eluent supplemented with 10 mM imidazole (pH 8.0) was incubated with nickel affinity resin (Ni-NTA, Qiagen) for 2 h at 4°C. The resin was washed with wash buffer B (lysis buffer A plus 0.01% GDN and 30 mM imidazole), and the protein was eluted with elution buffer B (lysis buffer A plus 0.01% GDN and 300 mM imidazole). The eluent was concentrated and subjected to size-exclusion chromatography (SEC, Superose 6 Increase, 10/300, GE Healthcare) in a buffer containing 25 mM HEPES (pH 7.4), 150 mM NaCl, and 0.01% GDN. The peak fractions were pooled and concentrated to ∼9 mg/ml for the cryo-EM analysis. For the cSID1-dsRNA sample, 100 μM 50-bp dsRNA molecules (5-ggccgggggacgggcugggaugacagaagucgcuuggugcagaucgggac-3) (GenScript) were mixed with the cSID1 proteins for 30 min before grids preparation. The expression and purification of the cSID1 mutants were the same as for the wild-type cSID1.

### Cryo-EM data acquisition

Holey carbon grids (Quantifoil Au 300 mesh, R1.2/1.3) were glow-discharged in the Plasma Cleaner PDC-32G-2 (Harrick Plasma Company) with a vacuum for 2 min and mid force for 35 s. Aliquots (4 μl) of apo-cSID1 or cSID1-dsRNA proteins were placed on the glow-discharged grids, which were then blotted for 3 s and flash frozen in liquid ethane cooled by liquid nitrogen using Vitrobot Mark IV (Thermo Fisher Scientific) at 8°C and 100% humidity. The grids were loaded onto a 300 kV Titan Krios G3i electron microscope (Thermo Fisher Scientific Inc.) equipped with K3 Summit detector (Gatan) and GIF Quantum energy filter. Images were automatically collected using AutoEMation (27) in super-resolution mode at a nominal magnification of 81 000×, with a slit width of 20 eV on the energy filter. A defocus series ranging from −1.3 μm to −1.8 μm was used. Each stack was exposed for 2.56 s with an exposure time of 0.08 s per frame, resulting in a total of 32 frames per stack and the total dose was approximately 50 e^−^/Å^2^ for each stack. The stacks were motion corrected with MotionCor2 (28) and binned 2-fold, resulting in a pixel size of 1.0825 Å/pixel. Meanwhile, dose weighting was performed (29). The defocus values were estimated with Gctf (30).

### Image processing

Dose-weighted micrographs were used for contrast transfer function (CTF) estimation using Patch-CTF in cryoSPARC (31). Micrographs with CTF fitting resolution worse than 4.0 Å were excluded during manual curation. Initial particles were picked from good micrographs using blob picker in cryoSPARC (31) and 2D averages were generated. Final particle picking was done by template picker using templates from the 2D results. Particles were extracted with a box size of 256 pixels and cropped into 128 pixels to accelerate early-step calculation and the yielded particles were re-extracted for final refinement.

For apo-cSID1 complex dataset, 2 839 micrographs were collected and 2 131 439 particles were auto-picked using blob picker in cryoSPARC. After several rounds of 2D classification, 350 209 good particles from 34 classes with different view directions were selected for template picking and Ab-initio reconstruction to generate initial references with C2 symmetry imposed. Subsequently, Heterogeneous Refinement (C2 symmetry) was carried out on full-set 3 241 223 particles picked by template picker using the references generated from Ab-initio reconstruction. The yielded good particles were then re-extracted using Bin1 (binning factor of 1) parameters and subjected to Heterogeneous Refinement, Non-uniform Refinement and Local Refinement with C2 symmetry, finally generating a density map reported at an overall resolution of 2.21 Å using 762 464 particles.

The data processing workflow of cSID1-dsRNA complex dataset is similar with that of apo- cSID1 dataset. However, both C1 and C2 symmetry were imposed during Heterogeneous Refinement, Non-uniform Refinement and Local Refinement. Finally, density maps reported at 2.35 Å (C1 symmetry) and 2.21 Å (C2 symmetry) were reconstructed from 340 359 and 449 206 particles, respectively. Resolutions were estimated using the gold-standard Fourier shell correlation (FSC) 0.143 criterion (32). Local resolution variations were estimated in cryoSPARC.

### Model building and structure refinement

An initial structure model for cSID1 was generated by AlphaFold (33). The predicted ECD and TMD structures were docked into the density map and manually adjusted and re-built by COOT (34), respectively. Sequence assignment was guided mainly by bulky residues such as Phe, Tyr, Trp and Arg. Unique patterns of sequences were exploited for validation of residue assignment. For the ECD, the glycosylation sites and disulfide bonds also facilitate sequence assignment. For the cSID-dsRNA structure, the apo-cSID1 structure and a dsRNA structure were docked into the density map and manually adjusted and re-built by COOT, respectively. Structure refinements were carried out by PHENIX (35) in real space with secondary structure and geometry restraints. The statistics of the 3D reconstruction and model refinement are summarized in Supplementary Table S1.

### Electrophoretic mobility shift assay (EMSA)

FAM labeled 50-bp dsRNA (5-ggccgggggacgggcugggaugacagaagucgcuuggugcagaucgggac-3) was used. Increasing concentration of wild-type cSID1 or cSID1 mutants (0, 2, 4, 8, 12, 16, 24 μM) was incubated with 1 μM of FAM labeled 50-bp dsRNA at room temperature for 60 min. EMSA reaction buffer containing 25 mM Tris, pH 8.0, 25 mM NaCl. After incubation, the mixture was subjected to electrophoresis in 6% native acrylamide gels in 0.5× Tris–borate–EDTA buffer (pH8.3) under a voltage of 100 V for about 100 min. The gel was soaked by 1:1000 diluted Goldview dye for 30 min and then visualized using Tanon 1600 Gel Imaging System (Tanon). The dsRNA intensity was quantified using FIJI software. The fraction of dsRNA bound was determined from the background-subtracted signal intensities using the expression: bound/(bound plus unbound). The fraction of dsRNA bound in each reaction was plotted versus the concentration of cSID1 or cSID1 mutants. Experiment was repeated independently three times and data are plotted as mean ± s.d.

## Results

### Structural determination of cSID1 under two conditions

To achieve a better understanding of cSID1 recognition by dsRNA, we determined the cryo-EM structures of the apo-cSID1 and its complex with a 50-bp dsRNA. As cSID1 binds to dsRNA in a sequence-independent manner (16), the sequence of the 50-bp dsRNA is arranged in a random order.

The detailed protocols of the protein purification, sample preparation, cryo-EM data acquisition, and structural determination are presented in Materials and Methods, Supplementary Figures S1–S5, and Supplementary Table S1. The apo-cSID1, cSID1-dsRNA without symmetry (C1), and cSID1-dsRNA with C2 symmetry (C2) structures were determined at overall resolutions of 2.21, 2.35 and 2.21 Å, respectively (Supplementary Figures S2 and S3). Two reliable dsRNA densities with a same conformation were observed in the map of cSID1-dsRNA (C1), therefore, we used the cSID1-dsRNA (C2) (hereafter cSID1-dsRNA) for structural analysis. The 2.21 Å EM maps display excellent main chain connectivity and side chain densities of cSID1 (Supplementary Figure S4). Notably, the dsRNA sequence can be assigned based on several bases with good densities in the interface, despite most RNA region has lower densities (Supplementary Figure S5). In addition, two cholesterol-shaped lipids were inserted into the hydrophobic cleft formed by transmembrane helix (TM) 9 and TM10 (Supplementary Figures S5, S6A, B). Another two cholesterol-shaped lipids were positioned adjacent to the TM1 and TM3 (Supplementary Figures S5, S6A, B). Intriguingly, a POPC-shaped lipid was buried into the hydrophobic cavity formed by TM6-9 (Supplementary Figures S5, S6A, C).

### Overall structure of cSID1

**Figure 1.**
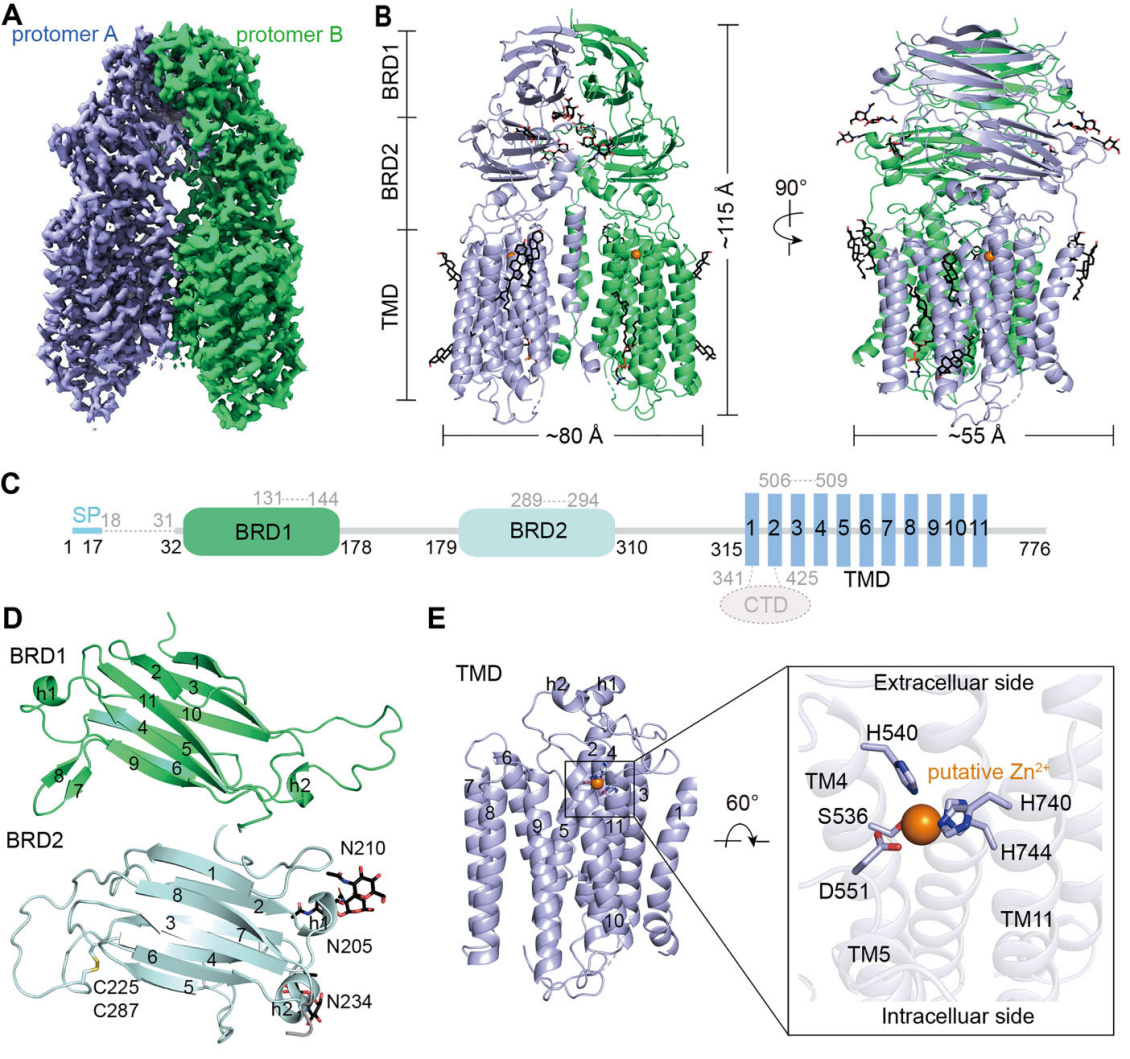
Overall structure of cSID1. (**A**) The overall EM density map of cSID1. cSID1 is a homodimer, and the protomers A and B are shown in limegreen and light blue, respectively. The map was contoured at 0.85. (**B**) Overall structure of cSID1. The N-linked glycans and lipids are displayed as black sticks. All structural figures were prepared in PyMOL (www.pymol.org). (**C**) A schematic diagram showing the domain organization of cSID1. SP, signal peptide; BRD, β-sheet rich domain; TMD, transmembrane domain. CTD, cytoplasmic domain. The dashed line indicates the invisible region in the structure of apo-cSID1. (**D**) The structures of BRDs. BRD1 contains 2 short α-helices and 11 β-strands. BRD2 contains 2 short α-helices and 8 β-strands. One disulfide bond is formed between C225 and C287 of BRD2, and three glycosylation sites are observed in BRD2. (**E**) The structure of TMD. The TMD contains 11 transmembrane helices containing a putative Zn^2+^-dependent catalytic core formed by three histidine residues, one aspartate residue, and one serine residue.

Akin to the structure of hSIDT2 (26), cSID1 also adopts a dimeric assembly through an extensive dimer interface that involved both the extracellular domain (ECD) and transmembrane domain (TMD) (Figure 1A,B). The overall structure of cSID1 had dimensions of approximately 80 Å × 55 Å × 115 Å (Figure 1B). The *Caenorhabditis elegans SID1* gene encodes a 776-amino acid protein with a signal peptide (residues 1–17), two β-strand rich domains (BRDs), a cytoplasmic domain (CTD) located between TM1 and TM2, and a TMD with eleven transmembrane segments (Figure 1C).

BRD1 residues 18–178 fold into 2 short α-helices and 11 β-strands, wherein β1, β3, β10, β5 and β6 are packed together and β2, β11, β4 and β9 are well ordered on the opposite side. β7 and β8 are nearly perpendicular to β6 and β9, respectively (Figure 1D). BRD2 residues 179–310-fold into 2 short α-helices and 8 β-strands with 3 glycosylation sites (Figure 1D). The β1, β8, β3 and β6 are packed together and β2, β7, β4 and β5 are well ordered on the opposite side. A disulfide bond is formed between C225 and C287 (Figure 1D). A Zn^2+^-dependent catalytic center is formed by three histidine residues (H740 and H744 in TM11 and H540 in TM4), S533 in TM4, and D551 in TM5 (Figure 1E), this H3-S-D motif is highly conserved among the CREST (ACER, progesterone adipoQ receptor (PAQR) receptor, Per1, SID1, and TMEM8) superfamily (36).

The dimer interface of cSID1 can be divided into three regions. The first region is formed by the β1, β3, β10, β5 and β6 of the two BRD1 molecules. The second region is mainly formed between the loop4–5 of the two BRD2 molecules. The third region is mainly formed between TM2 of one protomer with TM6 of the opposing protomer (Supplementary Figure S7).

Although cSID1 shares the same folding pattern with hSIDT1 and hSIDT2, dramatic conformational changes were found in the ECD, TM1, TM2 and TM6-9 regions when compared to the structures of hSIDT1 and hSIDT2 (Supplementary Figure S8), reflecting species specificity (Supplementary Figures S9 and S10).

### Overall structure of cSID1 bound to dsRNA

**Figure 2.**
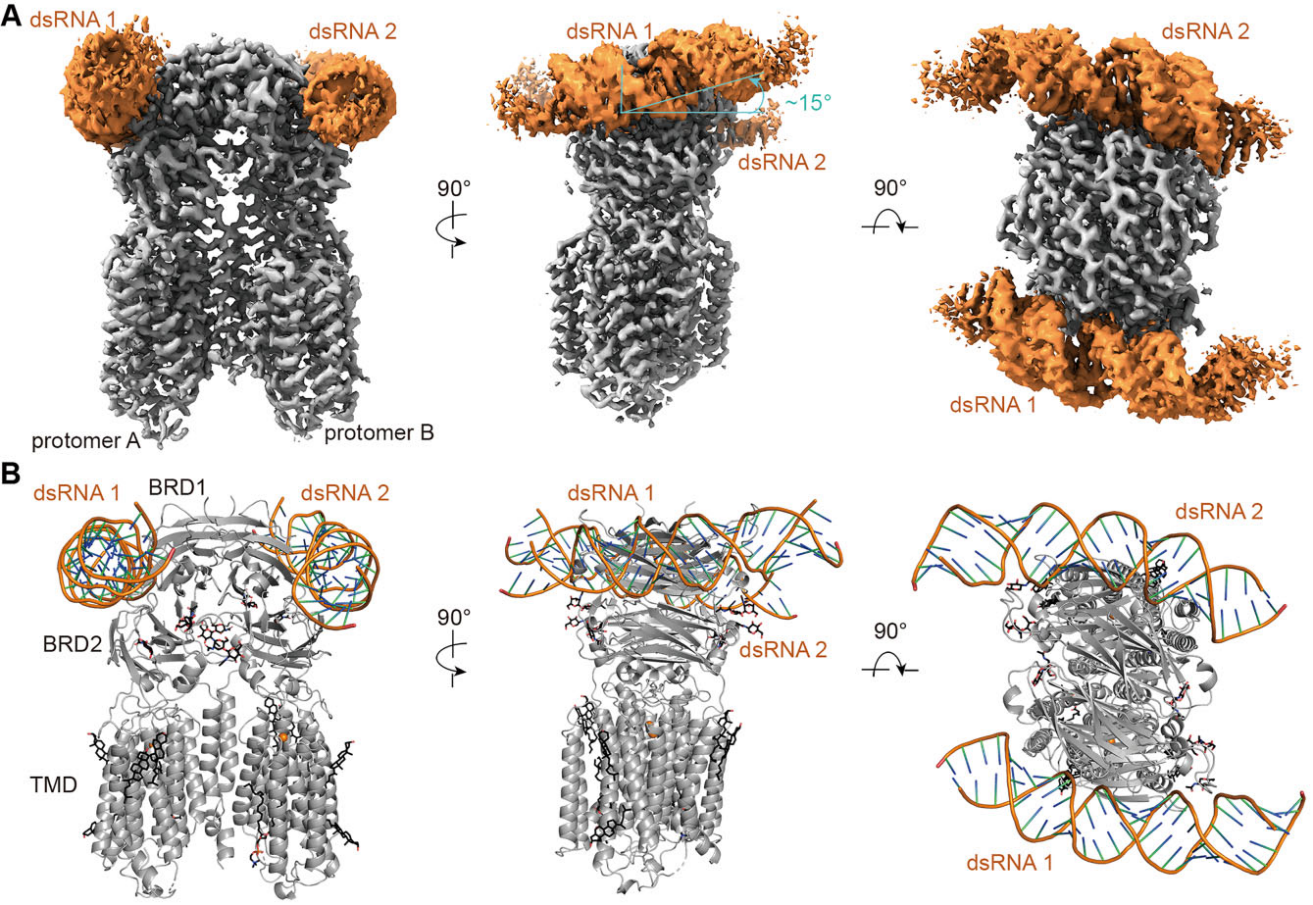
Overall structure of cSID1 bound to dsRNA. (**A**) The overall EM density map of cSID1-dsRNA complex. Each protomer binds to one dsRNA molecule. The dsRNA molecule is nearly parallel to the membrane plane only except the 5′-end region of the forward chain. (**B**) Overall structure of cSID1-dsRNA complex.

Each cSID1 protomer can bind to one dsRNA molecule, which is located at the interface between BRD1 and BRD2 (Figure 2A, B). The dsRNA molecule is mainly anchored by the BRD1 and the BRD2 acts as a scaffold placed below the dsRNA, stabilizing the interactions between dsRNA and BRD1 (Figure 2A, B). With the exception of the 5′-end of the forward chain, where the dsRNA molecule tilts at an angle of approximately 15° off the membrane plane, the dsRNA molecule is nearly parallel to the membrane plane (Figure 2A, B).

### dsRNA recognition by BRD1

**Figure 3.**
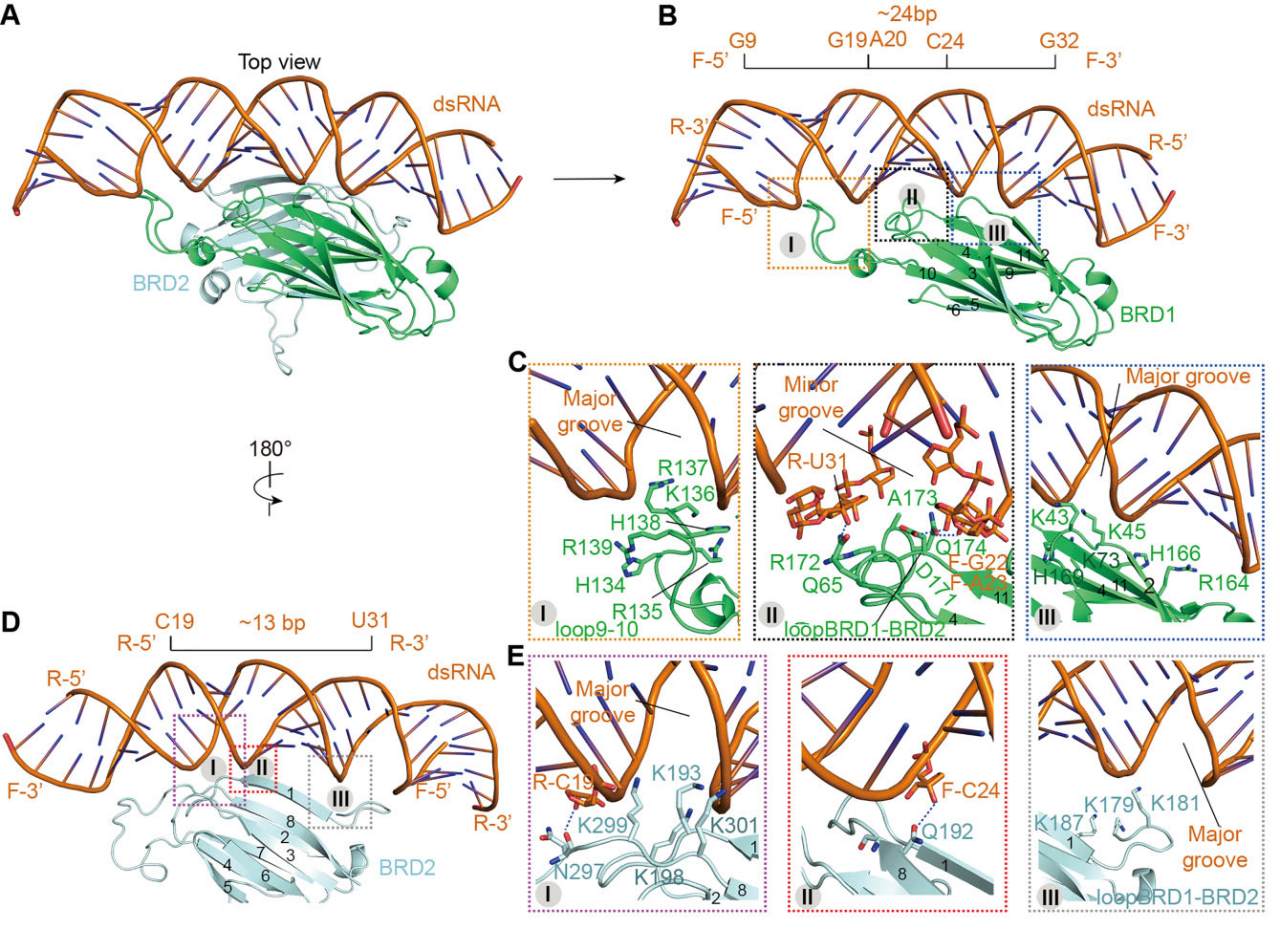
dsRNA recognition by the BRDs. (**A**) The dsRNA molecule is located at the interface between BRD1 and BRD2, and mainly anchored by the BRD1. The BRD2 acts as a scaffold placed below the dsRNA. (**B**) The binding surface between BRD1 and dsRNA. The binding surface on BRD1 can be divided into three interfaces, and about 24-bp region of the dsRNA molecule is involved in binding BRD1. (**C**) Left panel, the first interface is formed between loop9-10 of BRD1 and a major groove of dsRNA. The contact basic residues are indicated. Middle panel, the second interface is mainly formed between loopBRD1-BRD2 and loop 3–4 of BRD1 with a minor groove of dsRNA. Q65, D171 and Q174 form hydrogen bonds with the 2′-hydroxyl group of R-U31, F-G22, and F-A23, respectively. R, reverse chain. F, forward chain. Right panel, the third interface is formed between the phosphate backbone of a major groove formed by U21-G32 region of dsRNA with the loop1–2, β4 and β11 of BRD1. (**D**) The binding interface between BRD2 and dsRNA. The interface on BRD2 can be divided into three regions, and about 13-bp region of the dsRNA molecule is involved in binding BRD2. (**E**) Left panel, the first region is formed between loop 1–2 and loop 7–8 of BRD2 with the phosphate backbone of a major groove of dsRNA. N297 forms a hydrogen bond with the 2′-hydroxyl group of R-C19. Middle panel, the second region is formed by a hydrogen bond between Q192 and 2′-hydroxyl group of F-C24. Right panel, the third region is mainly formed between loopBRD1-BRD2 and a major groove of dsRNA.

The structure of cSID1-dsRNA reveals three surface patches on BRD1 that interact with dsRNA. The first interface is formed between the loop 9–10 (loop between β9 and β10) of BRD1 and a major groove of the dsRNA molecule, mainly mediated by extensive ionic interactions (Figure 3A–C, Supplementary Figure S11). Loop 9–10, a positively charged loop formed by H134, R135, K136, R137, H138 and R139, is inserted into the major groove formed by G9-A20 region of the dsRNA and contacts with the negatively charged phosphate backbone (Figure 3C, left panel, Supplementary Figure S11A). The second interface is mainly formed between the loopBRD1-BRD2 (loop between BRD1 and BRD2) and a minor groove formed by G19-C24 region of the dsRNA (Figure 3B). D171 and Q174 form a hydrogen bond with the 2′-hydroxyl group of G22 and A23 in the forward chain (Figure 3C, middle panel), respectively. In addition, Q65 in the loop3-4 also forms a hydrogen bond with the 2′-hydroxyl group of U31 in the reverse chain (Figure 3C, middle panel). R172 contacts with the phosphate backbone (Figure 3C, middle panel, Supplementary Figure S11B). The third interface is mainly formed between the phosphate backbone of a major groove formed by U21-G32 region of dsRNA with the loop1-2, β4, and β11 of BRD1, a region rich in positively charged residues (Figure 3C, right panel, Supplementary Figure S11B). About 24-bp region of the dsRNA molecule is involved in binding of BRD1 (Figure 3B). No interactions between bases of dsRNA and residues of cSID1 was found, reflecting the sequence-independent binding properties of cSID1.

### dsRNA recognition by BRD2

The interface between dsRNA molecule and BRD2 can be also divided into three regions (Figure 3D, Supplementary Figure S11). The first region is mainly formed between the K193 and K198 in the loop 1–2 and K299 and K301 in the loop 7–8 of BRD2 with the phosphate backbone of a major groove formed by A23-G32 region of dsRNA molecule (Figure 3E, left panel, Supplementary Figure S11C). In addition, N297 in the loop7-8 form a hydrogen bond with the 2′-hydroxyl group of C19 in the reverse chain (Figure 3E, left panel). The second region is formed between the Q192 in β1 and the 2′-hydroxyl group of C24 in the forward chain by a hydrogen bond (Figure 3E, middle panel). The third region is formed between the K187 in β1 and K179 and K181 in the loopBRD1-BRD2 with the phosphate backbone of C29-U31 in the reverse chain (Figure 3E, right panel, Supplementary Figure S11D). Similarly, the bases of dsRNA have no effect on the BRD2 binding.

### The electrostatic potential surface of cSID1

**Figure 4.**
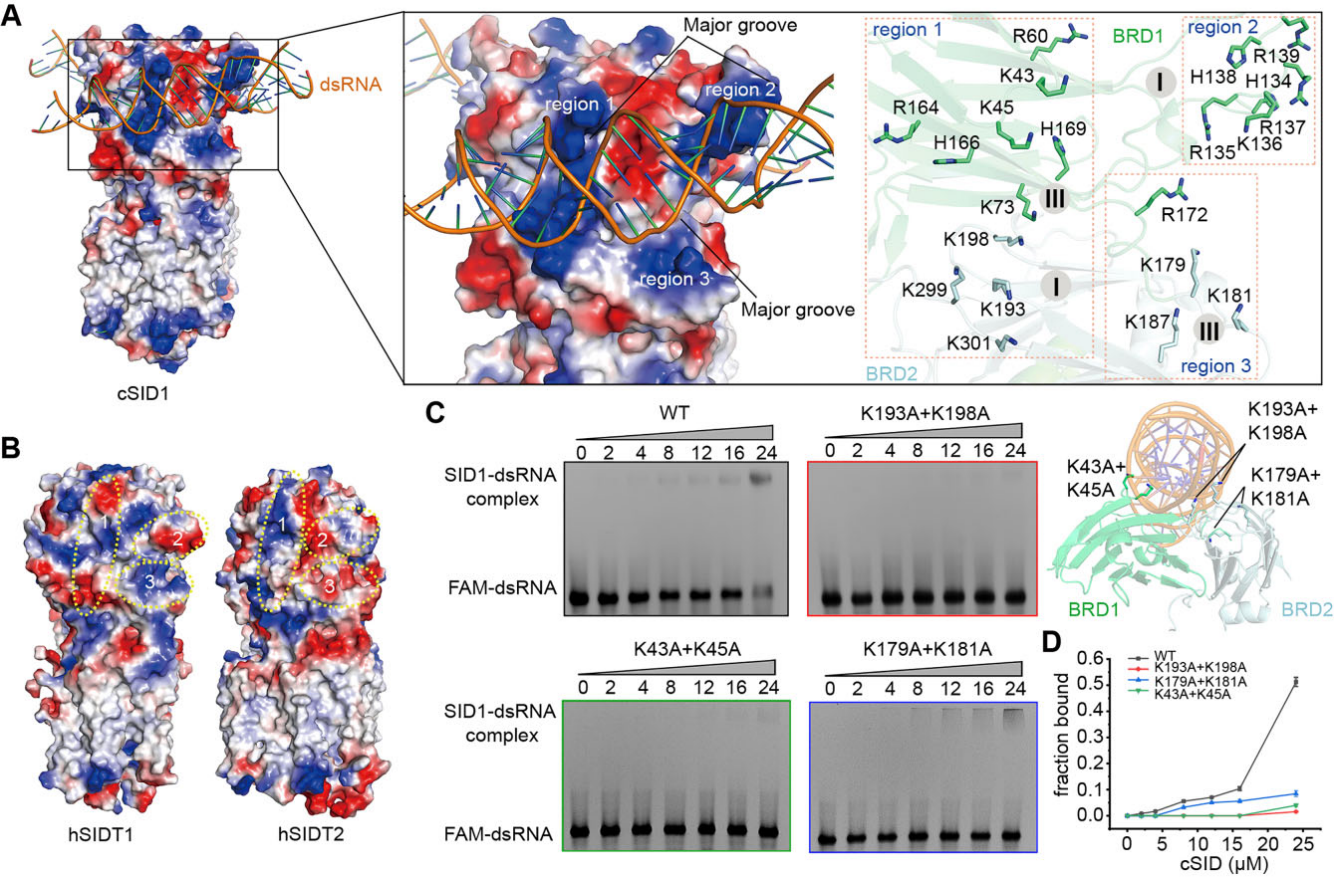
The electrostatic potential surface of cSID1 for binding dsRNA. (**A**) Three basic regions are fitted perfectly into three major grooves of dsRNA. The contact basic residues in these regions are indicated. (**B**) The hSIDT1 and hSIDT2 are devoid of these three basic regions. (**C**) EMSA results of wild-type cSID1 and cSID1 mutants in binding FAM labeled 50-bp dsRNA. Upper right panel indicates the locations of the three mutants. All the mutants can attenuate the dsRNA binding ability. (**D**) Quantitation of the fluorescent signal in figure C. Data are plotted as mean ± s.d.

The electrostatic potential surface shows there are three positively charged regions in the ECD, matching perfectly with three major grooves of the dsRNA molecule (Figure 4A). The region 1 is formed by the positively charged residues in the third binding interface of BRD1 and in the first binding interface of BRD2, representing the largest basic region in the ECD (Figures 3C, E, 4A). The region 2 is formed by the positively charged residues in the first binding interface of BRD1 (Figures 3C,4A). The region 3 is formed by the positively charged residues in the third binding interface of BRD2 (Figures 3C, 4A). The basic regions 1 and 2 act as two hooks for anchoring dsRNA molecule, and the basic region 3 serves as a scaffold to further stabilize the dsRNA binding. In contrast, the hSIDT1 and hSIDT2 are devoid of this structural feature (Figure 4B, Supplementary Figure S9 and S10), reflecting their apparent lower binding affinities for dsRNA (16,26).

To further consolidate the structural discovery of the interfaces between cSID1 and dsRNA, we designed three combined mutants to test their dsRNA binding ability by electrophoretic mobility shift assay (EMSA). Compared to the wild type cSID1, all these three mutants obviously disturbed their interactions with dsRNA (Figure 4C, D). Notably, the K43A + K45A and K193A + K198A mutants were able to almost completely eliminate dsRNA binding (Figure 4C, D), confirming that these positively charged residues are the contact sites for dsRNA binding.

### Conformational alterations of cSID1 upon dsRNA binding

**Figure 5.**
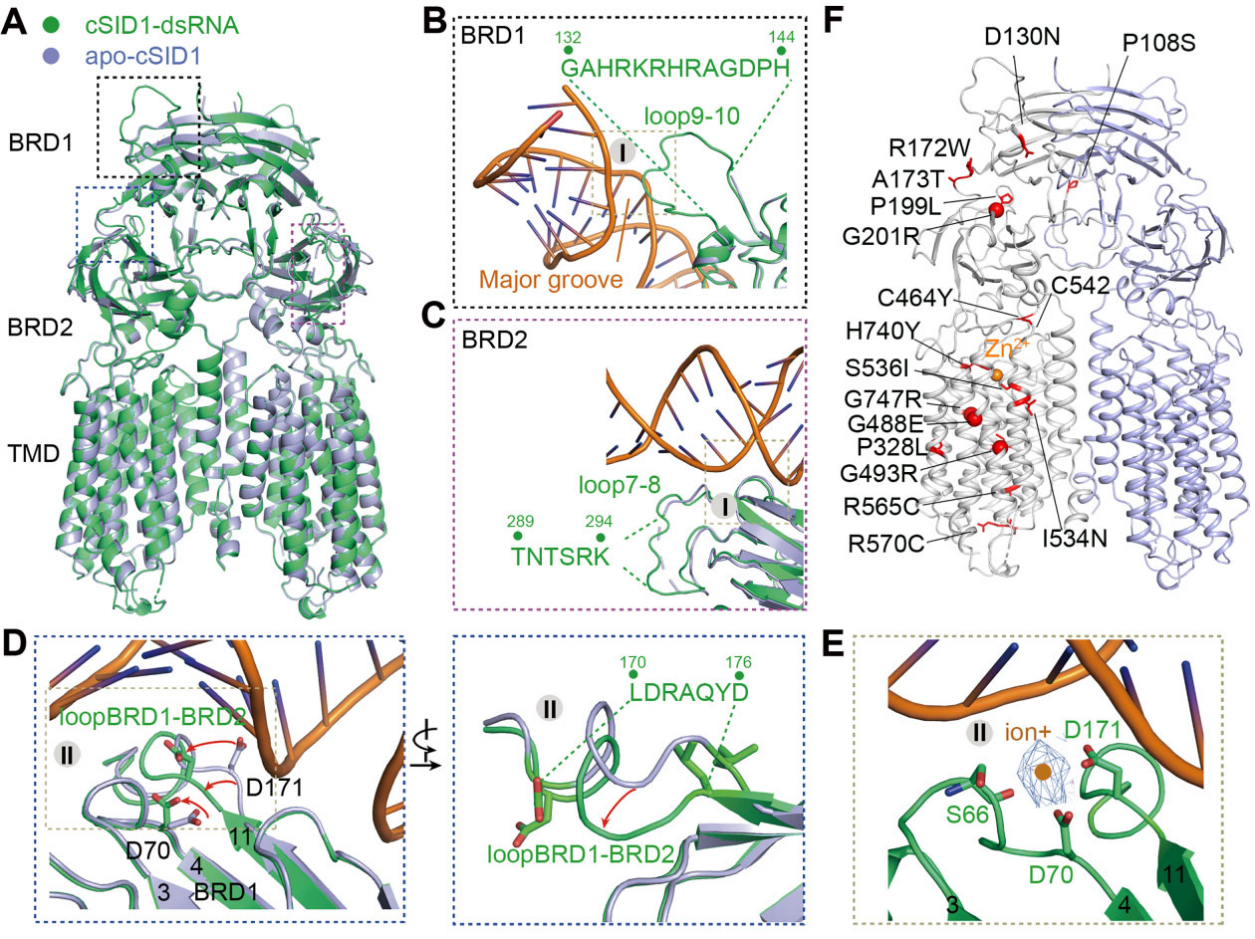
Conformational changes in cSID1 upon dsRNA binding and structural mapping of the missense mutations onto cSID1. (**A**) Comparison of the structure of apo-cSID1 with that of cSID1-dsRNA. Three dsRNA binding interfaces on ECD show significant conformational changes, whereas other regions are the same. (**B**) The loop 9–10 of BRD1 is resolved upon dsRNA binding. (**C**) Loop 7–8 of BRD2 is visible in the map of cSID1-dsRNA. (**D**) Loop 3–4 of BRD1 and loopBRD1-BRD2 underwent obvious conformational changes. Right panel, loopBRD1-BRD2 shows an obvious movement toward the putative positively charged ion that displayed in (E). (**E**) S66, D70 and D171 coordinate a putative positively charged ion. (**F**) Structural mapping of the missense mutations identified by a previous genetic screen onto cSID1.

Compared to the structure of apo-cSID1, three regions in the binding interfaces display dramatical conformational alterations whereas other regions of cSID1 remain unchanged (Figure 5A). The loop 9–10 in the first binding interface of BRD1 is resolved in the map of cSID1-dsRNA, indicating this region is stabilized by binding of dsRNA (Figure 5B). Similarly, the loop 7–8 that positioned adjacent to the first binding interface of BRD2 is also visible in the map of cSID1-dsRNA (Figure 5C). Notably, D70 in the loop 3–4 of BRD1 and D171 in the loopBRD1-BRD2 underwent a significant conformational change in the structure of cSID1-dsRNA, coordinating a putative positively charged ion in cooperation with S66 (Figure 5D,E). Accordingly, the L170-D176 region of loopBRD1-BRD2 (corresponding the second binding interface of BRD1) underwent a significant movement toward the putative positively charged ion (Figure 5D). This result indicates that the ion promotes the interactions between BRD1 and the negatively charged phosphate backbone of dsRNA.

### Structural interpretation of the missense mutations identified by a previous genetic screen

A previous genetic screen had identified dozens of loss-of-function mutations in cSID1 (2,37). Structural determination of cSID1 affords the opportunity to map a large number of the point mutations (Figure 5F). The mutations P108S and D130N are in the vicinity to the dimer interface, suggesting that they may affect the dimer stability. The mutations R172W and A173T are located in the second binding interface between BRD1 and dsRNA (Figures 3C, 5F), indicating that they may lessen dsRNA binding. Specifically, the A173T may provide steric hindrance between BRD1 and dsRNA (Figure 3C). C464 is situated at the interface of ECD and TMD, where it forms a disulfide bond with C452 (Figure 5F). The C464Y mutation may cause destabilization of this region. Our previous study revealed that the human SIDT2 has a Zn^2+^-dependent catalytic core formed by a H_3_-D-S motif and can hydrolyze C18 ceramide (38), whose lipid hydrolytic activity is required for uptake of dsRNA (39). The catalytic core would be disrupted by the S536I and H740Y mutations. Furthermore, the G488E, G493R, C559Y and G747R mutations are situated within the substrate-binding cavity (Figure 5F). The substitution with bulky residues may induce steric hindrance for substrate binding, reflecting a correlation between lipid hydrolytic activity and dsRNA transport.

## Discussion

The SID-1 family protein is a putative dsRNA channel or transporter that plays essential roles in diverse physiological processes, including systemic RNA interference (3), uptake and transfer of microRNA molecules (9), DNA and RNA autophagy (11,12), and antiviral innate immune response (8,10). However, the functional mechanism of SID1 family remains elusive. Recently, our group firstly reported the structure of hSIDT2 (26), and another two groups subsequently reported the structures of hSIDT1 (38,39), however, the structure of the hSIDT1/2-RNA complex was failed to be obtained. In this manuscript, we firstly report the structure of cSID1-dsRNA complex. As shown in Figures 3 and 4 and Supplementary Figure S11, the recognition of dsRNA by ECD is mainly mediated by extensive ionic interactions between the phosphate backbone of dsRNA and the basic residues of ECD. Intriguingly, Q65, D171, Q174, Q192 and N297 can form hydrogen bonds with the 2′-hydroxyl group of dsRNA (Figures 3 and 4). In addition, the particularly important regions for BRD1/2-dsRNA binding are three major grooves (Figure 4), which are narrowest for dsRNA, wider for DNA–RNA, and widest for dsDNA (40). Taken together, these structural features enable cSID1 to discriminate between dsRNA and dsDNA.

In principle, cSID1 would bind to different sub-segments of dsRNA as it binds to dsRNA in a sequence-independent manner. In fact, no additional stable conformation was observed during 3D classification. Although dsRNA sequence can be assigned based on several bases with good densities, we cannot rule out the possibility that the EM densities of dsRNA are an average of different sub-segments of dsRNA due to the poor densities for most RNA region (Supplementary Figure S5).

Similar to hSIDT2, cSID1 possesses a highly conserved Zn^2+^-dependent catalytic core and a large cavity within the TMs (Supplementary Figure S12), suggesting that SID1 may also potentially be involved in ceramide or phospholipase hydrolysis (26,39). In our earlier investigation, we superimposed the structure of hSIDT2 obtained by cryo-EM with that predicted by AlphaFold and detected substantial conformational changes in the TM6-9 and the loop between TM10 and helix 1 (h1) (hereafter loop 10-h1), whereby loop 10-h1 is positioned directly above the Zn^2+^-dependent catalytic core and TM6-9 is located at its lateral, indicating that they may have significant physiological significance (26). Subsequently, a POPC-shaped density was observed in the hydrophobic cleft formed by TM8–TM10 in the structure of SIDT1 E555Q mutant, suggesting that the substrate may enter the catalytic pocket via a lateral access route (39), wherein TM9 and TM10 serve as the gate (Supplementary Figure S12B). Interestingly, a POPC-shaped lipid was discovered in the hydrophobic cavity formed by TM6–9 and two cholesterol-shaped lipids were buried into the hydrophobic cleft formed by TM9–TM10 in this study (Supplementary Figure S7), raising the possibility that POPC and cholesterol play significant roles in controlling the lipid hydrolytic activity of cSID1.

Notably, no nucleic acid conduction pathway is observed in SID1 family proteins with a dimeric architecture (26), suggesting that SID1 family is not a dsRNA channel. Since we were unable to determine the structure of the hSIDT2-RNA complex in our previous investigation, we hypothesized that the SID1 family would function as a transporter based on the functional data from earlier literature (26). In this study, the structure of cSID1-dsRNA complex clearly showed that dsRNA molecule is anchored at the external surface of ECD and nearly parallel to the membrane plane (Figure 2), it appears to be impossible for a transporter to transport dsRNA with different length across membrane bilayer by the alternating-access mechanism. It has been reported that SIDT2 can interact with adaptor protein complexes AP-1 and AP-2 (41), which are heterotetramers that select cargo for inclusion into transport vesicles (42). Furthermore, knock-down of Clathrin, which encodes a protein participating in endocytosis, impaired the SIDT1-mediated dsRNA uptake (39). Also, SIDT2 can associate with apolipoprotein A1 (apoA1) and facilitate apoA1 secretion in hepatocytes (22). The lipid hydrolytic activity of SID1 family may also play important role in trimming the membrane lipids, facilitating the fission and fusion of membranes (39,43,44). It seems like that the uptake of dsRNA by SID1 family via a receptor-mediated endocytosis mechanism, wherein SID1 acts as the dsRNA receptor. However, the precise mechanism for the cSID1-mediated dsRNA internalization needs to be further investigated. In sum, our study represents a major step toward mechanistic understanding of SID1 family-mediated dsRNA uptake.

## Supplementary Material

gkae395_Supplemental_File

## Data Availability

The atomic coordinates and electron microscopy density maps of the three structures have been deposited in the PDB (http://www.rcsb.org) and the Electron Microscopy Data Bank (EMDB https://www.ebi.ac.uk/pdbe/emdb/). The accession numbers are as follows: apo-cSID1 (PDB: 8XBS; EMDB: EMD-38227) and cSID1-dsRNA (PDB: 8XC1; EMDB: EMD-38236). All other data are available from the corresponding authors upon reasonable request.
