# Supplementary material for: Structural basis for double-stranded RNA recognition by SID1
Source: Nucleic Acids Res. 2024 May 14;52(11):6718–27. doi: 10.1093/nar/gkae395 (PMC11194109; doi:10.1093/nar/gkae395)
Supplement: gkae395_Supplemental_File [file gkae395_supplemental_file.pdf]

Supplementary Data for

Structural basis for double-stranded RNA recognition by SID1

Runhao Wang[1#], Ye Cong[2#*], Dandan Qian[1*], Chuangye Yan[2*], Deshun Gong[1*]

Correspondence to:

congye@mail.tsinghua.edu.cn; qiandd@nankai.edu.cn; yancy2019@tsinghua.edu.cn; gongds@nankai.edu.cn.

This PDF file includes

Supplementary Figure S1 to S12

Supplementary Tables S1

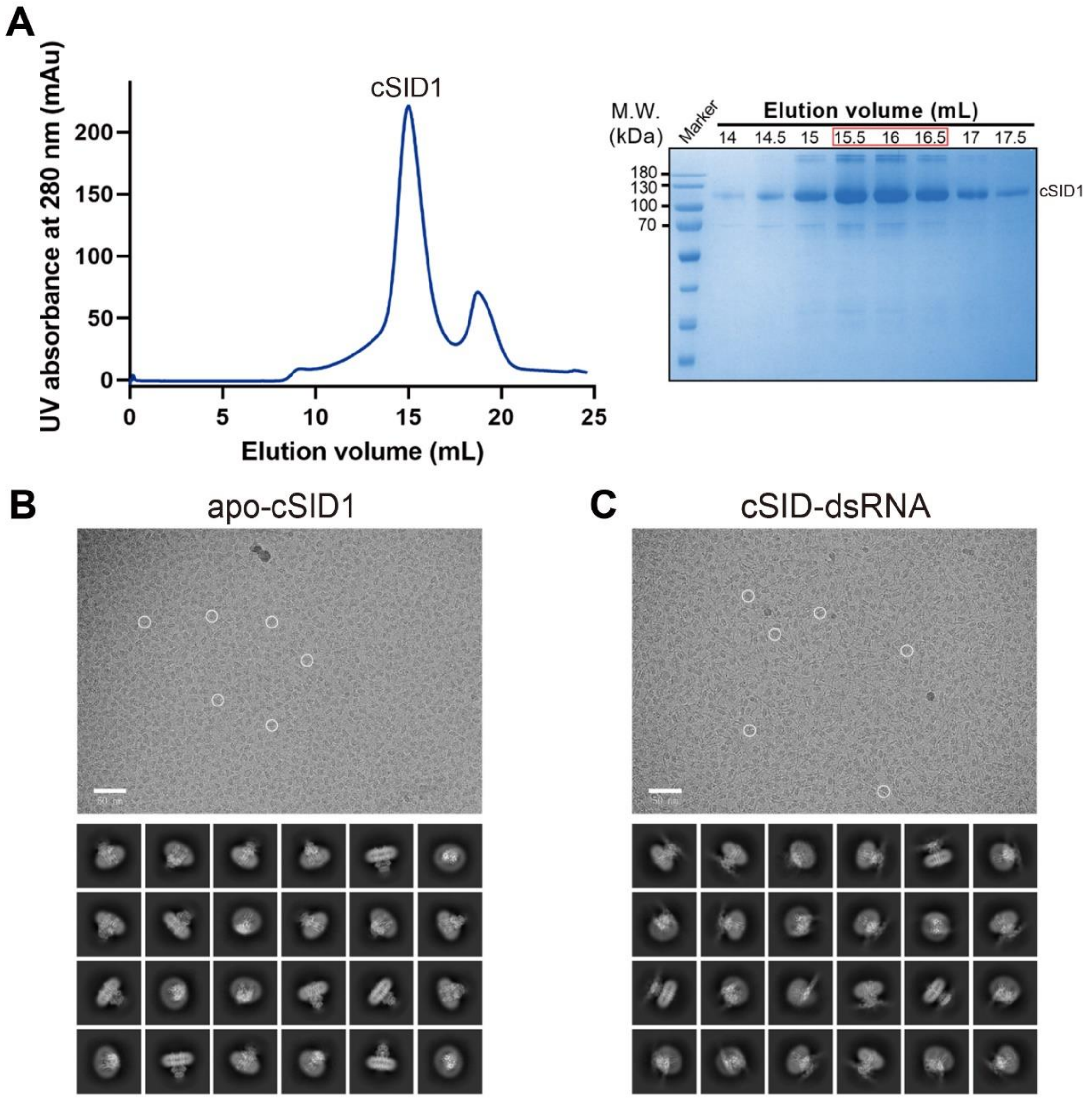

Supplementary Figure S1 | Protein purification and cryo-EM analysis of cSID1. **(A)** The cSID1 protein was subjected to size exclusion chromatography (SEC) and visualized by Coomassie blue staining. kDa, kilodaltons. M, marker. (**B)** Representative cryo-EM micrograph and 2D class averages of the apo-cSID1 sample. **(C)** Representative cryo-EM micrograph and 2D class averages of the cSID-dsRNA sample.

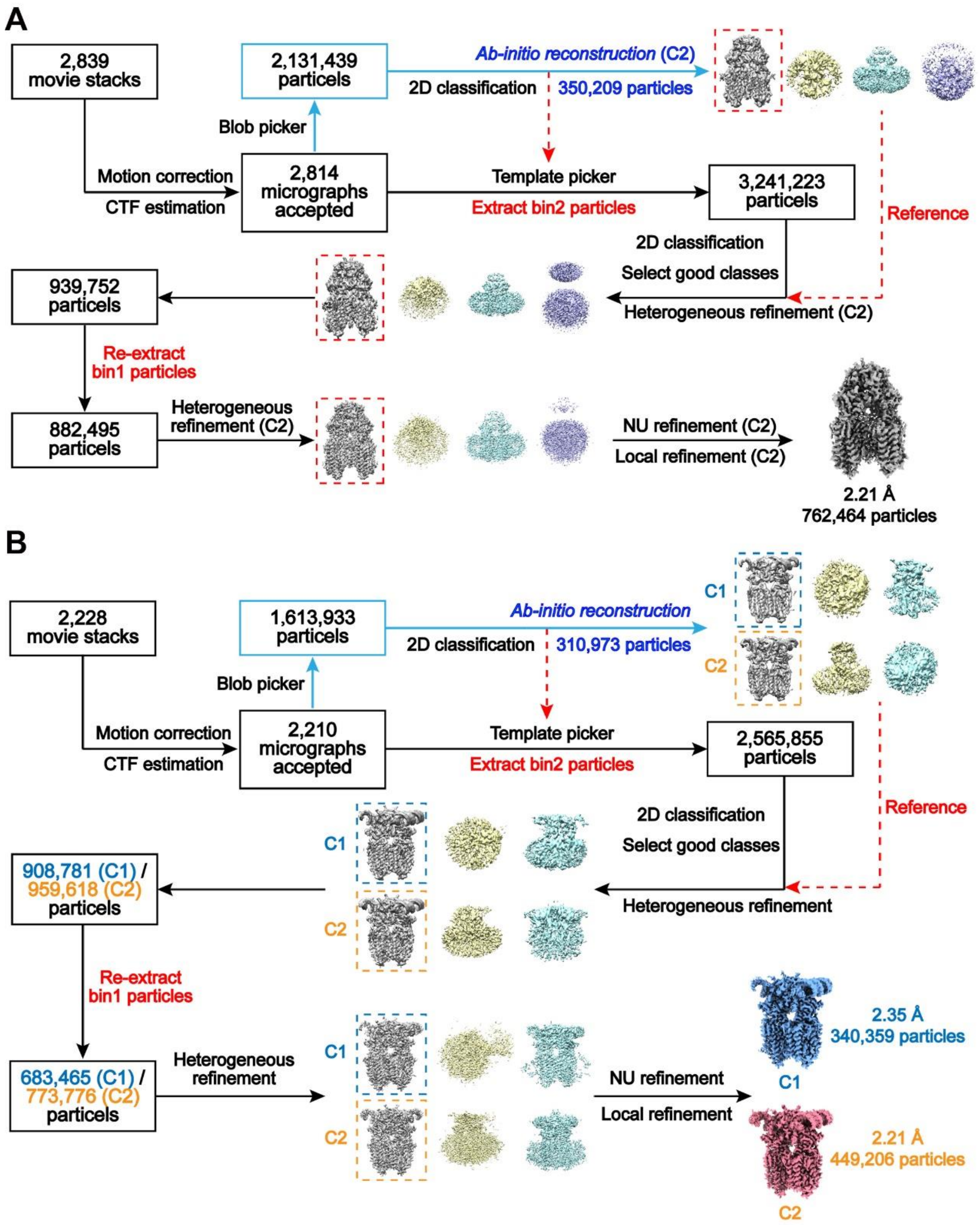

Supplementary Figure S2 | Flowchart for cryo-EM data processing. Please refer to Materials and methods for details.

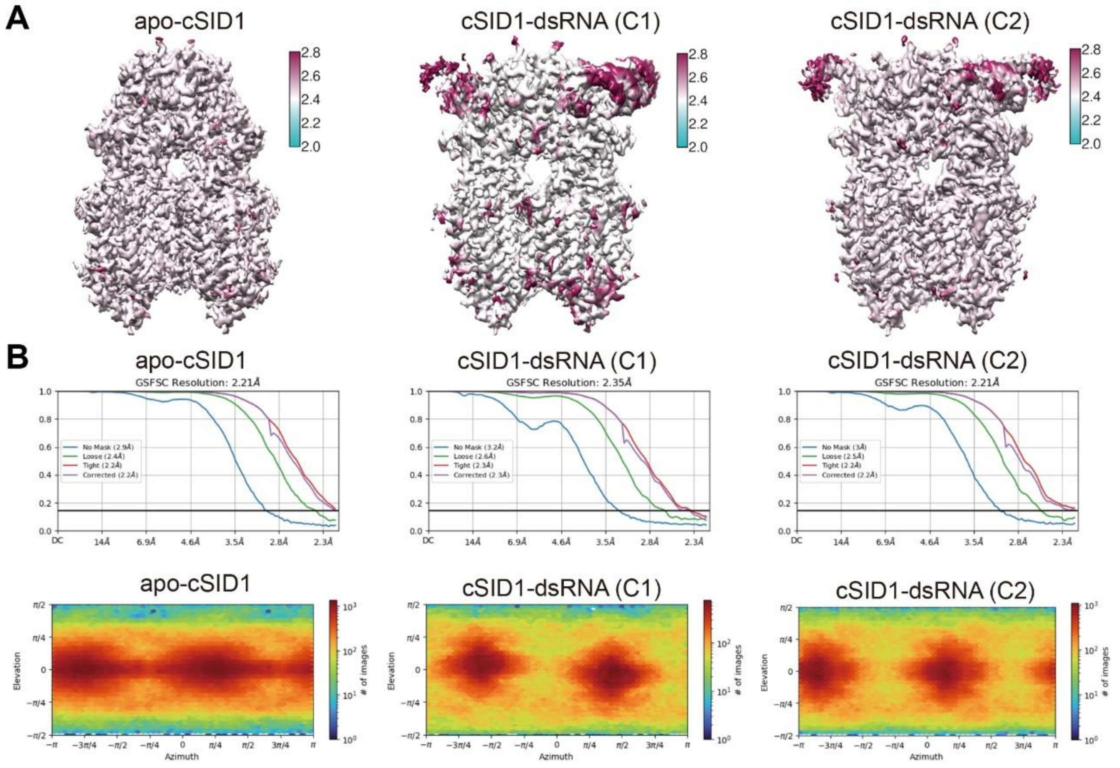

Supplementary Figure S3 | Local resolution maps, resolution estimation, and angular distribution of the three reconstructions. (A) The local resolution maps of the three reconstructions. **(B)** Gold-standard Fourier shell correlation curves and angular distribution of the particles used for the final reconstructions of the three reconstructions.

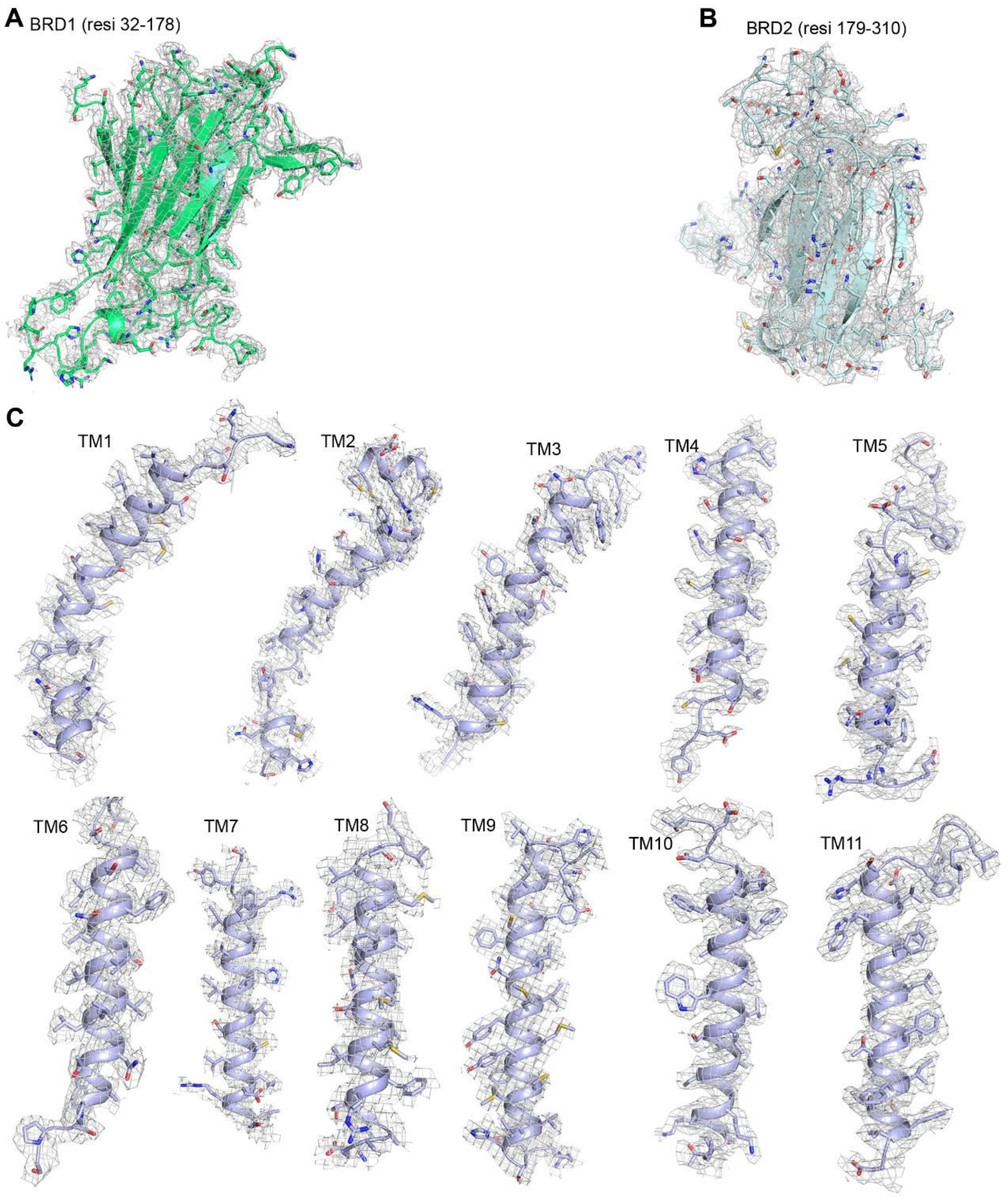

Supplementary Figure S4 | Representative local EM maps of cSID1. (A) The EM map of the BRD1. **(B)** The EM map of the BRD2. **(C)** The EM maps of the eleven TM helices.

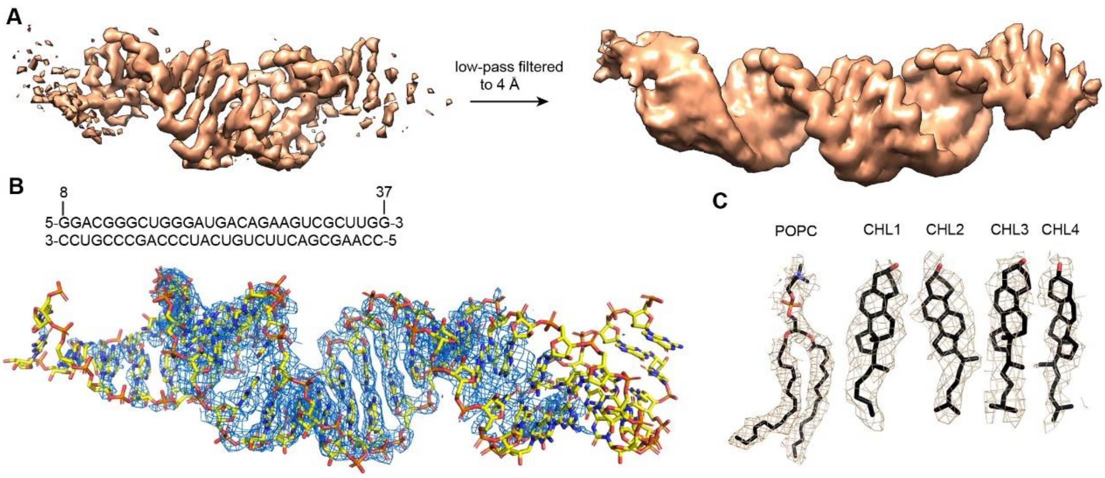

Supplementary Figure S5 | EM map of dsRNA and lipids. (A) EM map of dsRNA. A low-pass filtered map is indicated. (**B)** The atomic model for the G8-G37 region of the 50-bp dsRNA was successfully built. (**C)** The EM densities of the POPC-shaped and cholesterol-shaped lipids.

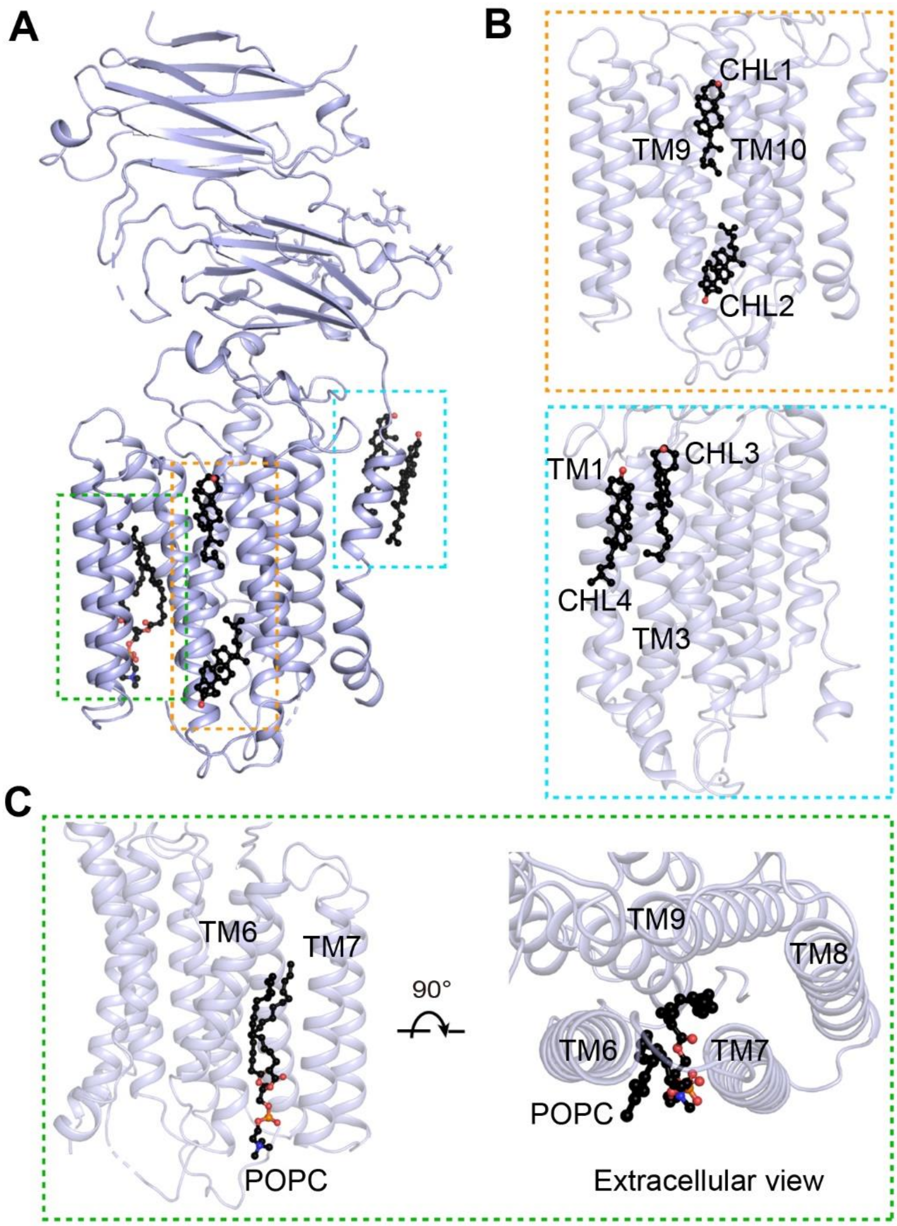

Supplementary Figure S6 | The locations of lipids on cSID1. (A) One POPC-shape and four cholesterol-shape lipids were observed on the cSID1. **(B)** Cholesterol (CHL) 1 and CHL2 were inserted into the hydrophobic cleft formed by TM9 and TM10. CHL3 and CHL4 were positioned adjacent to the TM1 and TM3. (**C)** POPC is buried into a cavity formed by TM6-9.

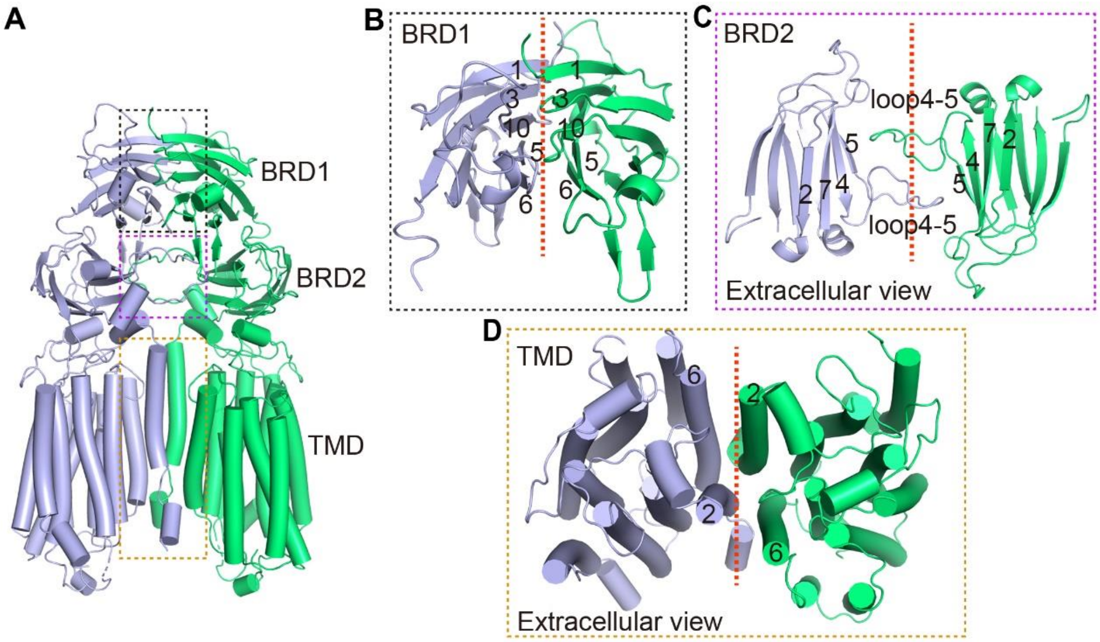

Supplementary Figure S7 | Dimer interface of cSID1. (A) The dimer interface of cSID1 can be divided into three regions. **(B)** The first region is formed by the β1, β3, β10, β5, and β6 of the two BRD1 molecules. (**C)** The second region is mainly formed between the loop4-5 of the two BRD2 molecules. **(D)** The third region is mainly formed between TM2 of one protomer with TM6 of the opposing protomer.

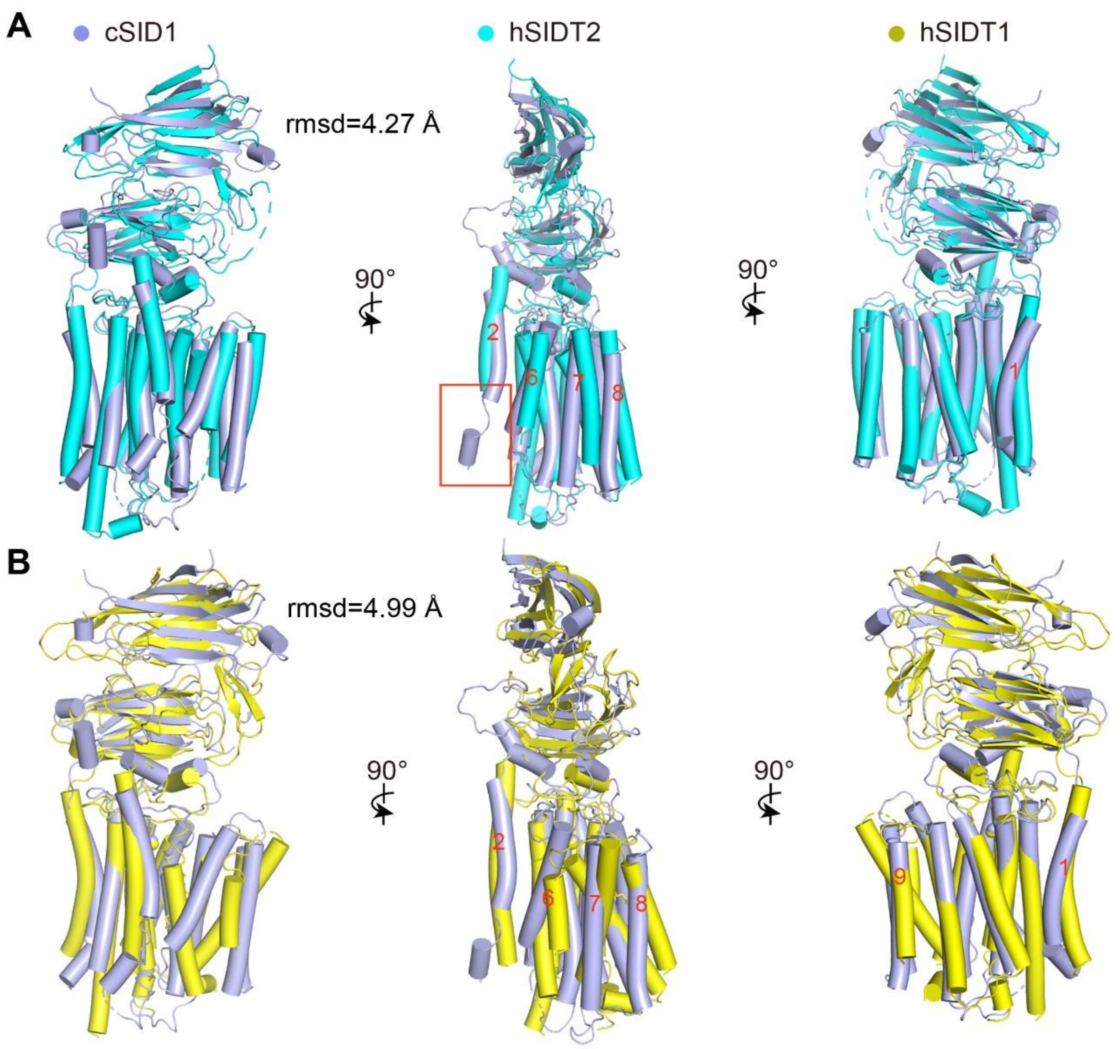

Supplementary Figure S8 | Comparison of the structure of cSID1 with those of hSIDT1 and hSIDT2. (A) Compared to the structure of hSIDT2, significant conformational changes were observed in the ECD, TM1, TM2, and TM6-9 of cSID1. **(B)** Similarly, compared to the structure of hSIDT1, dramatic conformational alterations were found in the ECD, TM1, TM2, and TM6-9 of cSID1.

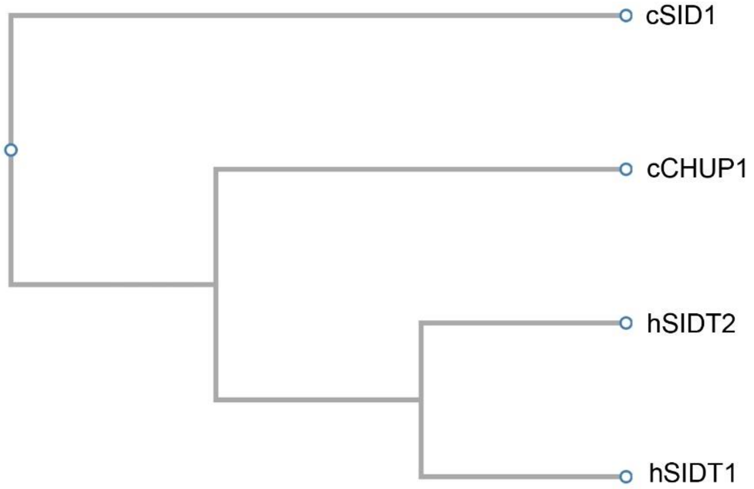

Supplementary Figure S9 | Phylogenetic tree of SID1 family. h, *homo sapiens*; c, *C. elegans*; CHUP1, tag-130/cholesterol uptake associated protein 1.

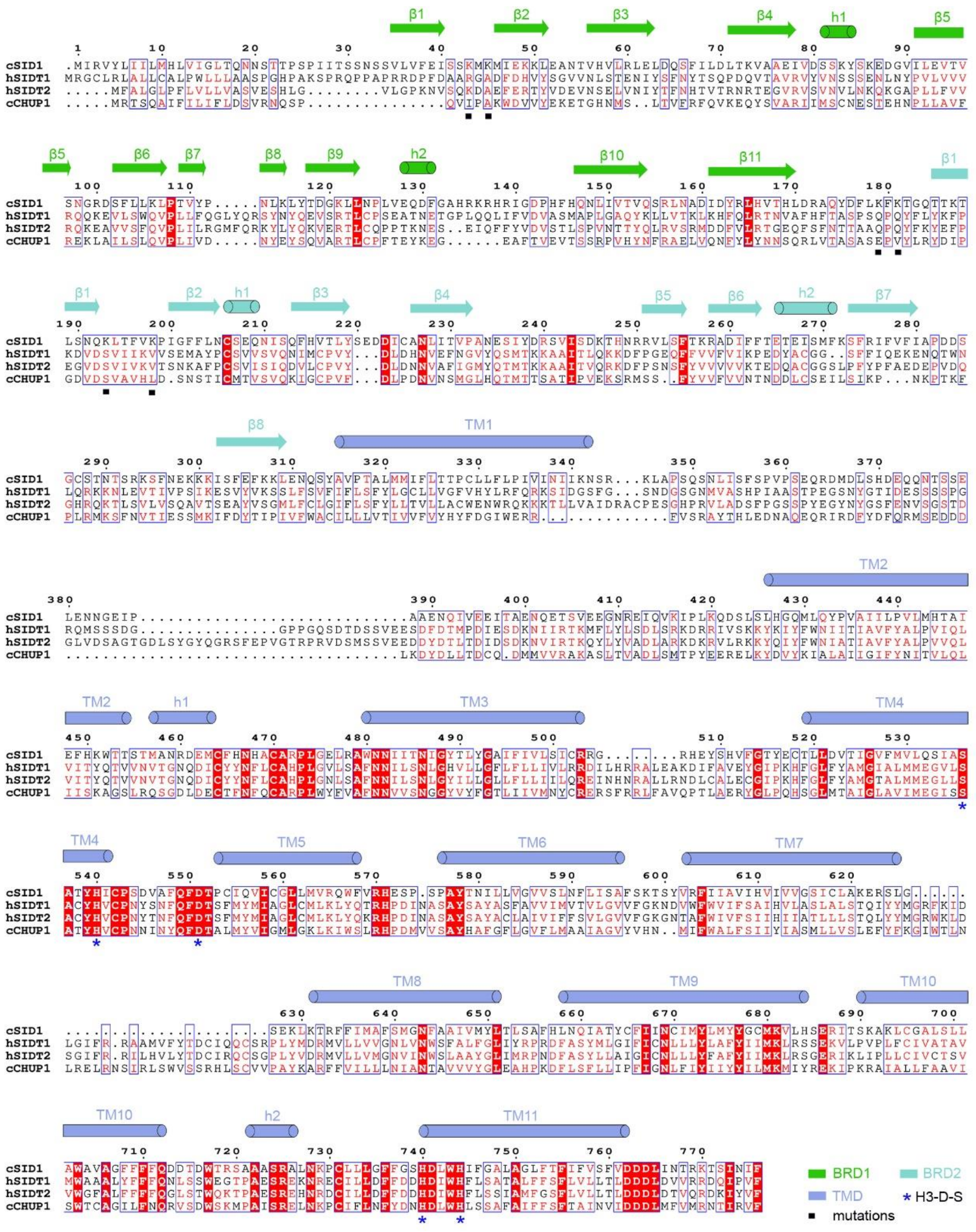

Supplementary Figure S10 | Sequence alignment of cSID1 with its homologues. The sequences were aligned using ClustalW. The secondary structural elements of cSID1 are indicated above the sequence alignment and domains are colored the same as the structures shown in the main text figures. h, *homo sapiens*; c, *C. elegans*. The blue stars indicate the catalytic Zn^{2+}-binding site. The black boxes indicate the mutations for EMSA assay.

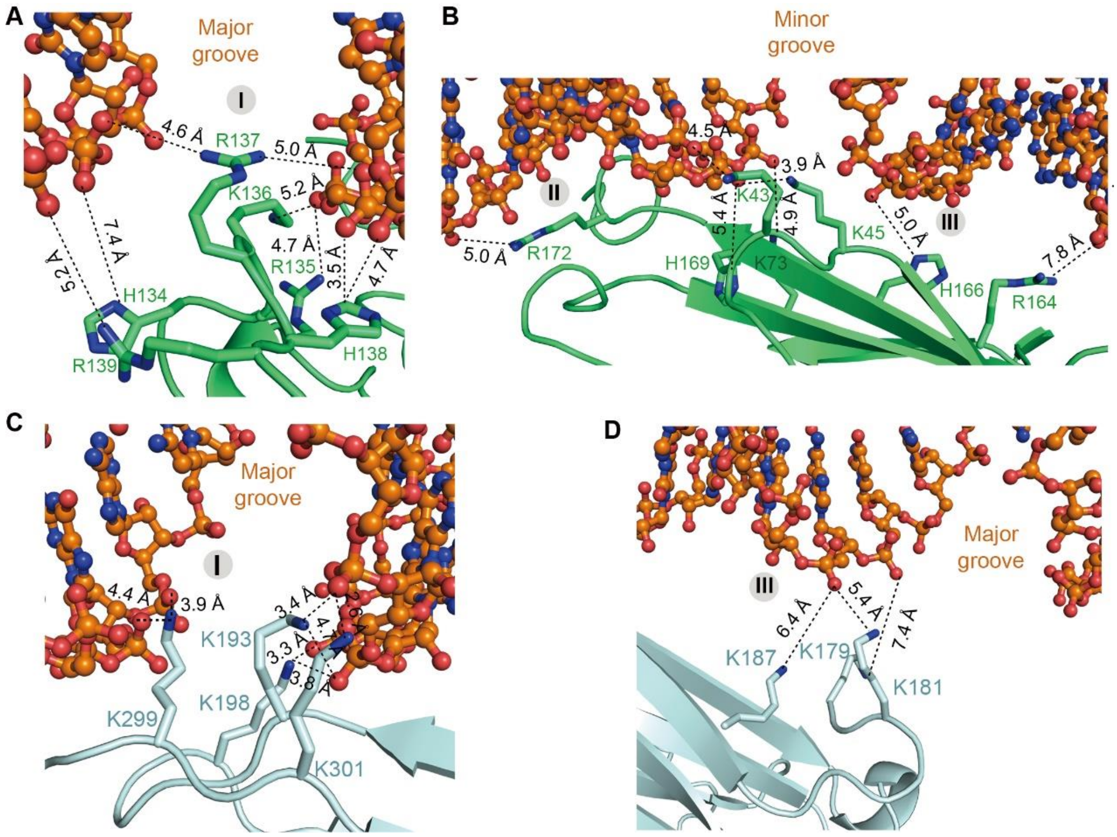

Supplementary Figure S11 | Possible ionic interactions between positively charged residues and negatively charged phosphate backbone. (A-D) The distances between the positively charged residues and phosphate are indicated.

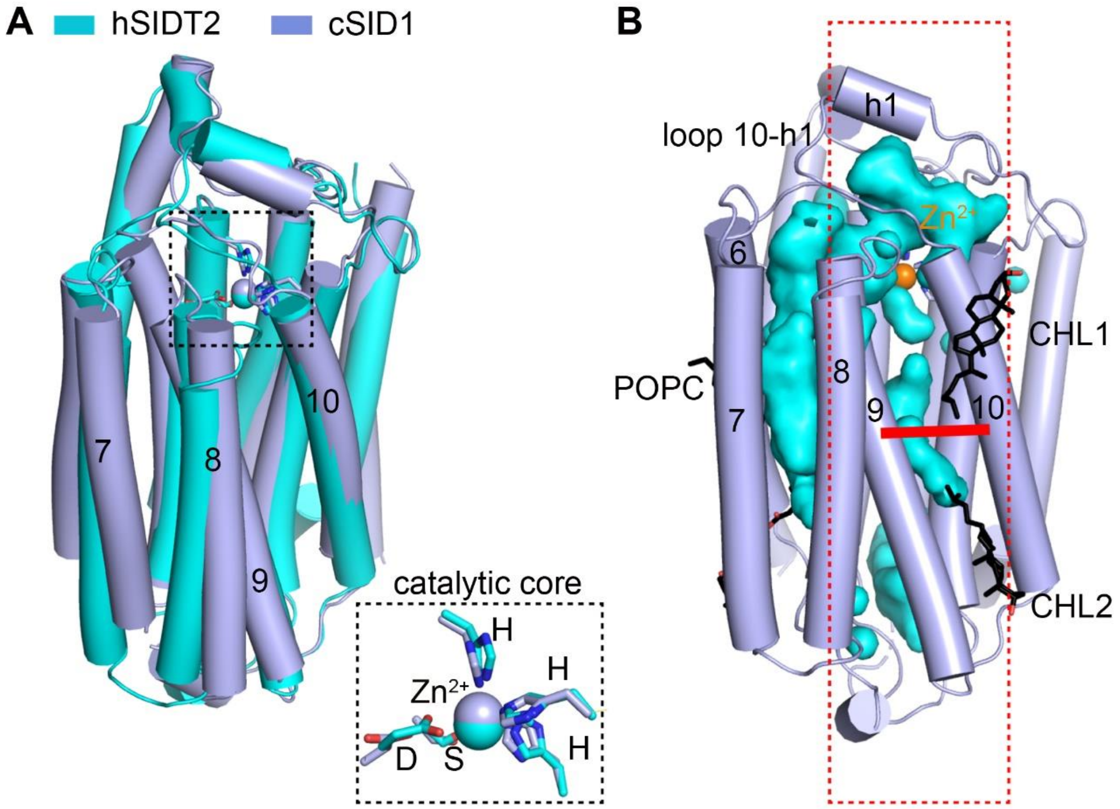

Supplementary Figure S12 | Comparison of the TMD of cSID1 with that of hSIDT2 and the large cavity within the TMD of cSID1. (A) cSID1 has a similar Zn^{2+}-dependent catalytic core with that of hSIDT2. **(B)** Akin to the hSIDT2, a large cavity is also observed within the TMD of cSID1. Red dashed box indicates the large cavity around the catalytic core. Red line indicates that TM9 and TM10 serve as the gate for substrate entry.

Supplementary Table S1 | Cryo-EM data collection, refinement and validation statistics

	apo-cSID1	cSID1-dsRNA	cSID1-dsRNA
**Data collection and processing**			
Magnification	81,000	81,000	81,000
Voltage (kV)	300	300	300
Electron exposure (e^{-}/Å^2)	50	50	50
Defocus range (μm)	-1.3 to -1.8	-1.3 to -1.8	-1.3 to -1.8
Pixel size (Å)	1.0825	1.0825	1.0825
Symmetry imposed	C2	C1	C2
Movies	2,839	2,228	2,228
Final particle images (no.)	762,464	340,359	449,206
Map resolution (Å)	2.21	2.35	2.21
FSC threshold	0.143	0.143	0.143
**Refinement**			
Model composition			
Non-hydrogen atoms	10658		13536
Protein residues	1282		1320
Nucleotide	-		120
Ligands	22		22
B factors (Å^2)			
Protein	45.9		63.4
Nucleotide	-		173.6
Ligand	65.5		79.1
R.m.s. deviations			
Bond lengths (Å)	0.007		0.005
Bond angles (°)	1.363		1.390
Validation			
MolProbity score	2.08		2.02
Clashscore	8.39		10.05
Poor rotamers (%)	3.17		2.05
Ramachandran plot			
Favored (%)	96.4		96.2
Allowed (%)	3.6		3.8
Outliers (%)	0.0		0
